# Analysis of Phenolic Metabolite Variations and Antioxidant Mechanisms in Foxtail Millet From Different Regions of Shanxi

**DOI:** 10.1002/fsn3.70778

**Published:** 2025-08-10

**Authors:** Xuan Zhao, Luhong Yang, Yaoru Li

**Affiliations:** ^1^ Shanxi Normal University School of Life Sciences Taiyuan China

**Keywords:** antioxidant, foxtail millet, phenolic metabolites

## Abstract

To investigate the differences in phenolic metabolites of foxtail millet from different regions and analyze their antioxidant mechanisms, this study used Jingu 21 foxtail millet as the research subject. Ultrasonic‐assisted extraction technology, in vitro antioxidant assays, UPLC‐MS/MS, and molecular docking were employed to systematically compare the phenolic metabolite characteristics of Jingu 21 foxtail millet from two regions in Shanxi. The results showed that the polyphenols in foxtail millet exhibited strong antioxidant capacity, with the millet from Longhua County (LC) demonstrating superior antioxidant properties compared to that from Anze County (AC). Through widely targeted metabolomics analysis, 186 phenolic metabolites with significant differences in content were identified between LC and AC, of which 124 differential polyphenols showed high accumulation in LC. These phenolic compounds were primarily flavonoids, including key flavonoid components such as Tricin, Homoplantaginin, and Iristectorin A. KEGG pathway enrichment analysis revealed that folate biosynthesis (ko00790) and phenylpropanoid biosynthesis (ko00940) were the most significantly different metabolic pathways. Molecular docking results with antioxidant‐related key proteins indicated that differential polyphenols with binding energies ≤ −11 kJ/mol, such as Homoplantaginin, 6‐Hydroxykaempferol‐7‐O‐glucoside, Luteolin‐3′‐O‐glucoside, and Cynaroside, were all highly accumulated in LC. In vitro experiments validated these findings for Homoplantaginin and Iristectorin A. A series of research results demonstrated that the antioxidant activity of phenolic metabolites in foxtail millet is influenced by regional environments, and the superior antioxidant capacity of LC compared to AC is mainly attributed to its abundant flavonoid content.

## Graphical abstract

This study revealed significant regional differences in phenolic metabolites of Jingu 21 foxtail millet from Shanxi. Longhua millet (LC) exhibited superior antioxidant capacity due to higher accumulation of flavonoids (e.g., Homoplantaginin, Tricin). Metabolomics identified 186 differential metabolites, with folate and phenylpropanoid biosynthesis as key pathways. Molecular docking confirmed strong antioxidant potential (binding energy ≤ −11 kJ/mol) for LC‐enriched compounds. Environmental factors strongly influenced flavonoid content and antioxidant activity.

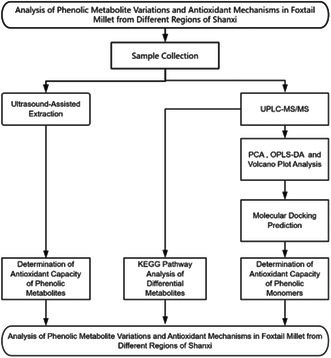

## 1 Introduction

Oxidation and antioxidant reactions play crucial roles in the body's metabolism. Under normal circumstances, these two processes remain coordinated and in a dynamic equilibrium, sustaining numerous physiological, biochemical, and immune reactions. When the body is exposed to various harmful stimuli, excessive production of highly reactive molecules such as reactive oxygen species (ROS) and reactive nitrogen species (RNS) occurs. These free radicals can directly or indirectly oxidize or damage DNA, proteins, and lipids, leading to gene mutations, protein denaturation, and lipid peroxidation, thereby inducing oxidative stress (OS). Oxidative stress is considered one of the most significant risk factors for human aging and various major diseases, including cancer, cardiovascular and cerebrovascular diseases, neurodegenerative disorders (e.g., Alzheimer's disease), and diabetes. Therefore, research on antioxidants holds great importance in safeguarding human health.

Foxtail millet, derived from the seeds of the grass species *Setaria italica* , possesses multiple potential health benefits (Gupta et al. 2012; Truswell 2002). As the sixth most produced cereal crop globally, foxtail millet is not only rich in dietary fiber, protein, and vitamins, but also attracts attention due to its unique phytochemical composition, among which phenolic compounds are regarded as the key bioactive constituents (Saleh et al. 2023). Phenolic compounds, a class of secondary metabolites widely present in plants, exhibit health‐promoting effects such as antioxidant, anti‐inflammatory, and antimicrobial properties.

Amid growing concerns over the safety of synthetic antioxidants, natural polyphenols in foxtail millet have garnered increased interest due to their high antioxidant efficacy, abundant sources, and safety (Saleh et al. 2023).

Northern China is a major foxtail millet‐producing region, with Shanxi Province standing out due to its unique geographical and climatic conditions and its cultivation of numerous high‐quality millet varieties (Wang, Qin, et al. 2018). However, the complex and diverse geographical environment of Shanxi may lead to significant differences in the composition and content of polyphenols in millet from different production areas.

Currently, there is limited systematic research on polyphenols in Shanxi foxtail millet, particularly in‐depth analyses of the differences in polyphenol metabolites and their antioxidant mechanisms across different production regions.

To address this gap, this study focuses on Jingu 21, a major foxtail millet cultivar in Shanxi. Samples were collected from two typical production areas—Longhua Town in Yicheng County and Anze County—to systematically analyze the antioxidant activity and mechanisms of millet polyphenols. By comparing the differences in polyphenol metabolites between millet from these two regions, the study explores how environmental factors impact millet quality from a polyphenol perspective. This research provides valuable insights for establishing quality evaluation systems and breeding superior millet varieties in Shanxi.

## 2 Research Content and Methods

### 2.1 Experimental Materials

The experimental variety used was Jingu 21, harvested in October 2023 from two production areas in Shanxi Province: Longhua Town, Yicheng County (111.95° E–111.87° E, 35.79° N–35.71° N), and the urban area of Anze County (112.5° E–112.35° E, 35.35° N–36.30° N). Uniform irrigation and fertilization practices were applied, and samples were collected at the same physiological maturity stage. The samples were labeled as LC (Longhua) and AC (Anze), respectively, and stored at low temperatures. All samples were prepared with three biological replicates.

### 2.2 Study on the Antioxidant Activity of Millet Phenolic Metabolites

#### 2.2.1 Ultrasonic‐Assisted Extraction of Millet Phenolic Metabolites

The two samples were ground into powder, and 1 g of each powder was weighed into a centrifuge tube. A solvent‐to‐material ratio of 1:30 was maintained by adding 30 mL of 50% ethanol as the solvent. The mixture was subjected to ultrasonic extraction at 50°C and 200 W for 1 h. After centrifugation, the supernatant was collected and stored at 4°C for further use.

#### 2.2.2 Determination of Antioxidant Capacity of Phenolic Metabolites

The LC extract was accurately diluted with 80% methanol to concentrations of 0, 50, 100, 150, and 200 μg/mL, while the AC extract was diluted to 0, 20, 40, 60, and 80 μg/mL for subsequent experiments.

DPPH Radical Scavenging Activity: The method was adapted from references (Rumpf et al. 2023; Wu et al. 2023) with slight modifications. The experimental setup included the following groups: control group (400 μL LC/AC extract + 400 μL 80% methanol solution), test group (400 μL LC/AC extract + 400 μL DPPH working solution), and blank group (400 μL 80% methanol solution + 400 μL DPPH working solution).

The mixtures were kept in the dark for 30 min, followed by centrifugation at 4000 rpm for 5 min. The absorbance of the reaction solution was measured at 517 nm using a microplate reader. The DPPH radical scavenging activity (RSA) was calculated using the following formula:

$$\mathrm{RSA}\;\left(\%\right)=\left(1-\left(A_{t}-A_{c}\right)÷A_{\text{blank}}\right)\times 100\%$$

where *A*_t_ is the absorbance of the test group, *A*_c_ is the absorbance of the control group, and *A*_blank_ is the absorbance of the blank group.

Inhibition of Fe^2+^‐Induced Lipid Peroxidation in Egg Yolk Lipoproteins: The method was modified from reference (Cao et al. 2004), using the thiobarbituric acid (TBA) colorimetric assay to determine the malondialdehyde (MDA) content, which reflects the extent of lipid peroxidation. The procedure was as follows: Add 0.4 mL of egg yolk suspension, 0.1 mL of sample at different concentrations, 0.4 mL of 25 mmol/L FeSO_4_, and 3.1 mL of PBS (0.1 mol/L, pH 7.45) into a centrifuge tube. Mix well. Incubate at 37°C with shaking for 15 min. Add 1 mL of 20% TCA and 1 mL of 0.8% TBA; then heat in a boiling water bath for 15 min. Cool the mixture, centrifuge at 3500 rpm for 10 min, and measure the absorbance of the MDA‐TBA complex in the supernatant at 532 nm. The inhibition rate was calculated using the formula:

$$\text{Inhibition rate}\;\left(\%\right)=\left(A_{1}-A_{\text{sample}}\right)/A_{1}\times 100\%$$

where *A*_1_ is the absorbance of the sample with 0.1 mL PBS buffer, and *A*_sample_ is the absorbance of the test sample.

All experiments were performed in triplicate; the results were plotted using Origin 2025.

### 2.3 Metabolite Qualitative and Quantitative Analysis

#### 2.3.1 Metabolites Extraction

After freeze‐drying, the samples were ground into a powder and dissolved in a specified amount of methanol. The supernatant was collected by centrifugation, filtered, and then subjected to instrumental analysis.

#### 2.3.2 UPLC Conditions

Chromatographic conditions: An Agilent SB‐C18 column (2.1 mm × 100 mm, 1.8 μm) was used. Mobile phase A consisted of ultrapure water (containing 0.1% formic acid), and mobile phase B was acetonitrile (containing 0.1% formic acid). The elution gradient for mobile phase B was as follows: 0–9 min, 5.

Mass spectrometry conditions: An electrospray ionization (ESI) source was used, with an ion source temperature of 550°C and an ion spray voltage (IS) of 5500 V (positive ion mode) or −4500 V (negative ion mode). Ion source gas I (GSI), gas II (GSII), and curtain gas (CUR) were set to 50, 60, and 25 psi, respectively, with collision‐induced dissociation parameters set to high. In the triple quadrupole (QQQ), each ion pair was scanned and detected based on optimized declustering potential (DP) and collision energy (CE).

#### 2.3.3 Data Analysis

Metabolites were identified using BMK Cloud Platform (Beijing), based on the Human Metabolome Database and Lipid Metabolites and Pathways Strategy. A triple quadrupole linear ion trap mass spectrometer was employed for sample detection under the specified parameters. T‐tests, OPLS‐DA analysis, PCA analysis, and Spearman correlation analysis were performed using R software. Differential metabolites (SCMs) were screened based on fold change (FC > 1), *p*‐value (*p* < 0.05), and VIP value (VIP > 1). Heatmap, volcano plot, and other analyses of SCMs were conducted using the Xiantao Academic Platform (xiantaozi.com) online software. Metabolic pathways were analyzed using KEGG Pathways. Molecular docking was performed using Autodock Vina and PyMOL to predict binding energy and interactions between differential metabolites and key antioxidant proteins.

### 2.4 Molecular Docking Prediction

The 2D structures of annotated polyphenol metabolites were downloaded from PubChem and converted to 3D structures using ChemOffice; then saved in .mol2 format. Target genes were searched in UniProt, and their protein IDs were used to obtain their 3D structures from the PDB database. Water molecules and small molecule ligands were removed from the 3D structures of target genes using PyMOL. Receptor and ligand files were prepared in .pdbqt format using AutoDock Vina; active pockets were defined, and docking was performed in Vina. The results were visualized using PyMOL.

### 2.5 Antioxidant Capacity Assay of Phenolic Monomers

Based on the molecular docking results, the characteristic phenolic monomers Homoplantaginin and Iristectorin A were selected to validate their antioxidant activity.

Inhibition of Fe^2+^‐Induced Lipid Peroxidation in Egg Yolk Lipoproteins: Homoplantaginin and Iristectorin A were accurately prepared in DMSO at concentrations of 10, 100, 500, 1000, 1500, and 2000 μg/mL, stored at 4°C in the dark, and used immediately in the experiments. The experimental method followed Section 2.2.2.

ABTS Total Antioxidant Capacity Assay: The total antioxidant capacity (T‐AOC) was measured according to Wu et al. (García et al. 2012; Wu et al. 2023), with slight modifications. Homoplantaginin was prepared in DMSO at concentrations of 10, 100, 200, 300, 400, and 500 μg/mL, while Iristectorin A was prepared at 10, 100, 500, 1000, 1500, and 2000 μg/mL. Both compounds were stored at 4°C in the dark and used immediately. A blank well containing 10 μL of distilled water and test wells containing 10 μL of sample solutions at various concentrations were set up. To all wells, 20 μL of oxidant application solution and 170 μL of ABTS working solution were added, mixed, and allowed to react at room temperature for 6 min. Absorbance was measured at 405 nm using a microplate reader. The ABTS‐scavenging ability was calculated using the following formula:

$$\text{ABTS}-\text{scavenging ability}\;\left(\%\right)=\left(A_{0}-A_{n}\right)/A_{0}\times 100\%$$

where *A*_0_ is the absorbance of the blank well, and *A*_n_ is the absorbance of the test well.

All experiments were performed in triplicate; the results were plotted using Origin 2025.

## 3 Results

### 3.1 Antioxidant Activity of Millet Phenolic Metabolite Extracts

**FIGURE 1:**
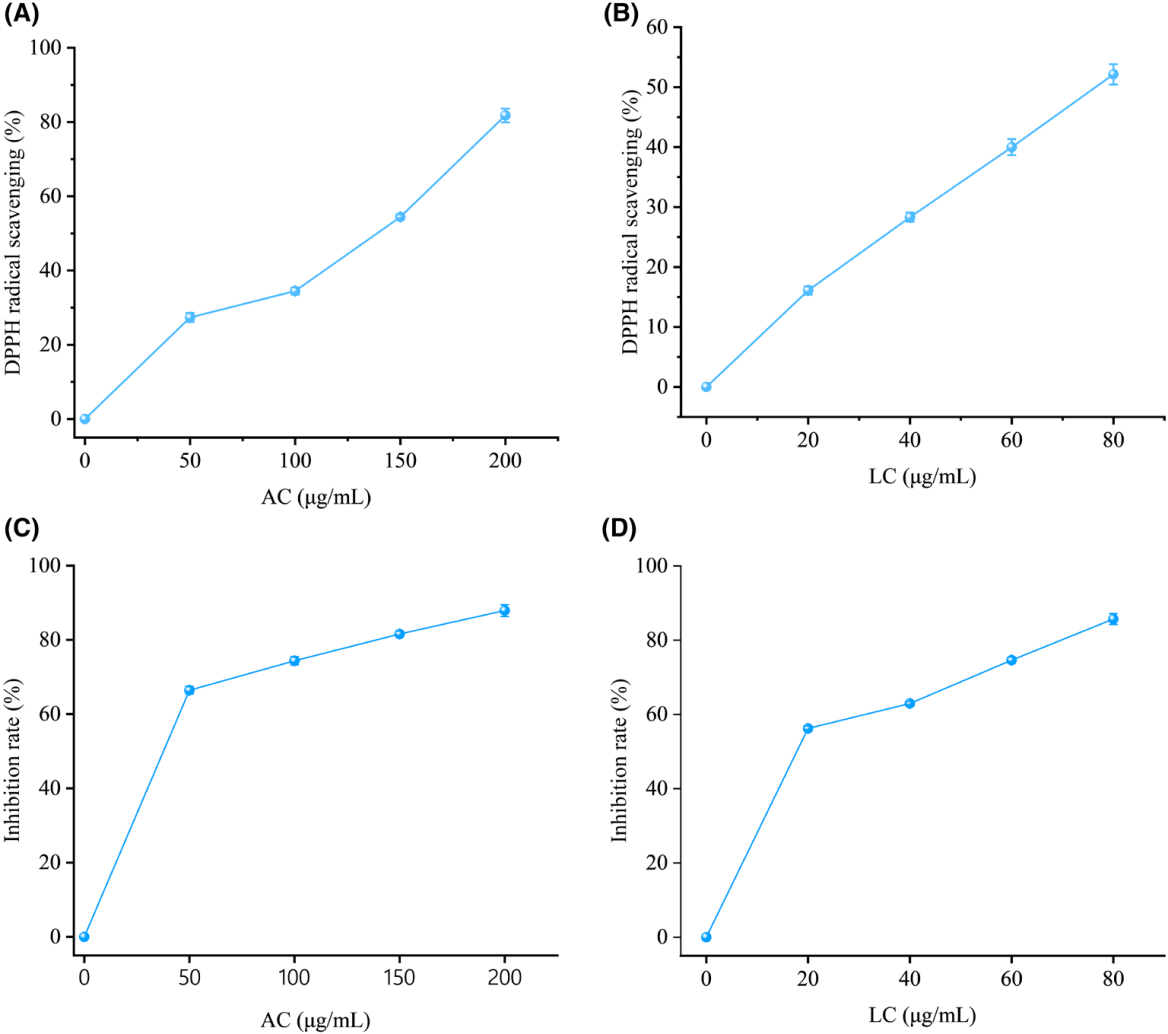
Antioxidant activity of phenolic metabolite extracts from samples. (A, B) represent DPPH radical scavenging assays; (C, D) represent anti‐lipid peroxidation activity assays.

As shown in Figure 1A,B, both LC and AC extracts exhibited DPPH radical scavenging capacity, which increased with higher sample concentrations. The AC extract reached a maximum scavenging rate of 28.36% ± 1.21% at 200 μg/mL, while the LC extract achieved a significantly higher maximum of 52.14% ± 1.70% at just 80 μg/mL.

The anti‐lipid peroxidation assay (Figure 1C,D) revealed that the inhibition rates of the LC and AC extracts on Fe^2+^‐induced lipid peroxidation in egg yolk lipoproteins were concentration‐dependent within a specific range. The AC extract exhibited a maximum inhibition rate of 88.24% ± 0.02% at 200 μg/mL; whereas the LC extract reached a comparable maximum inhibition rate of 86.33% ± 0.01% at 80 μg/mL.

### 3.2 Differential Analysis of Phenolic Metabolites in LC and AC

#### 3.2.1 PCA and OPLS‐DA Analysis

**FIGURE 2:**
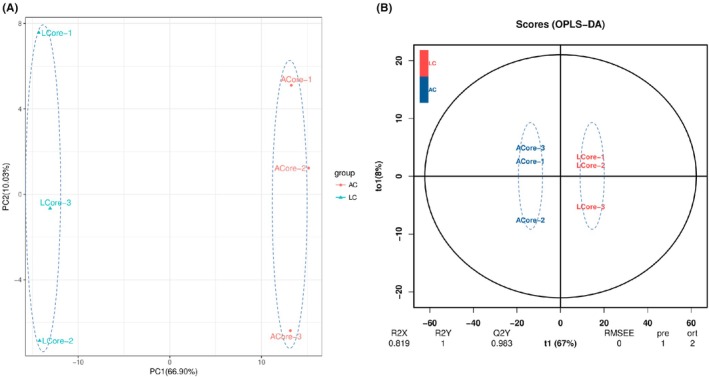
Statistical analysis results of millet samples from two production regions (A, B). PCA score plot (A) and (B) OPLS‐DA score plot (B). LCore and ACore represent Longhua‐produced millet and Anze‐produced millet, respectively.

PCA analysis of phenolic metabolites from the two regions (Figure 2A) showed that the first and second principal components accounted for 66.90% and 10.03% of the variance, respectively. The clear separation between LC (define if necessary) and AC (define if necessary) samples reflects significant differences in phenolic metabolite profiles between the regions.

OPLS‐DA (Figure 2B) demonstrated strong inter‐group discrimination (t1 = 67%, to1 = 8%). The model's evaluation parameters (all > 0.5, *Q* ^2^ *Y* > 0.9) confirmed its reliability for subsequent VIP‐based screening of differential phenolic metabolites. Both PCA and OPLS‐DA results confirmed robust and reproducible interregional metabolic differences.

#### 3.2.2 Differential Phenolic Metabolites in LC Versus AC

**FIGURE 3:**
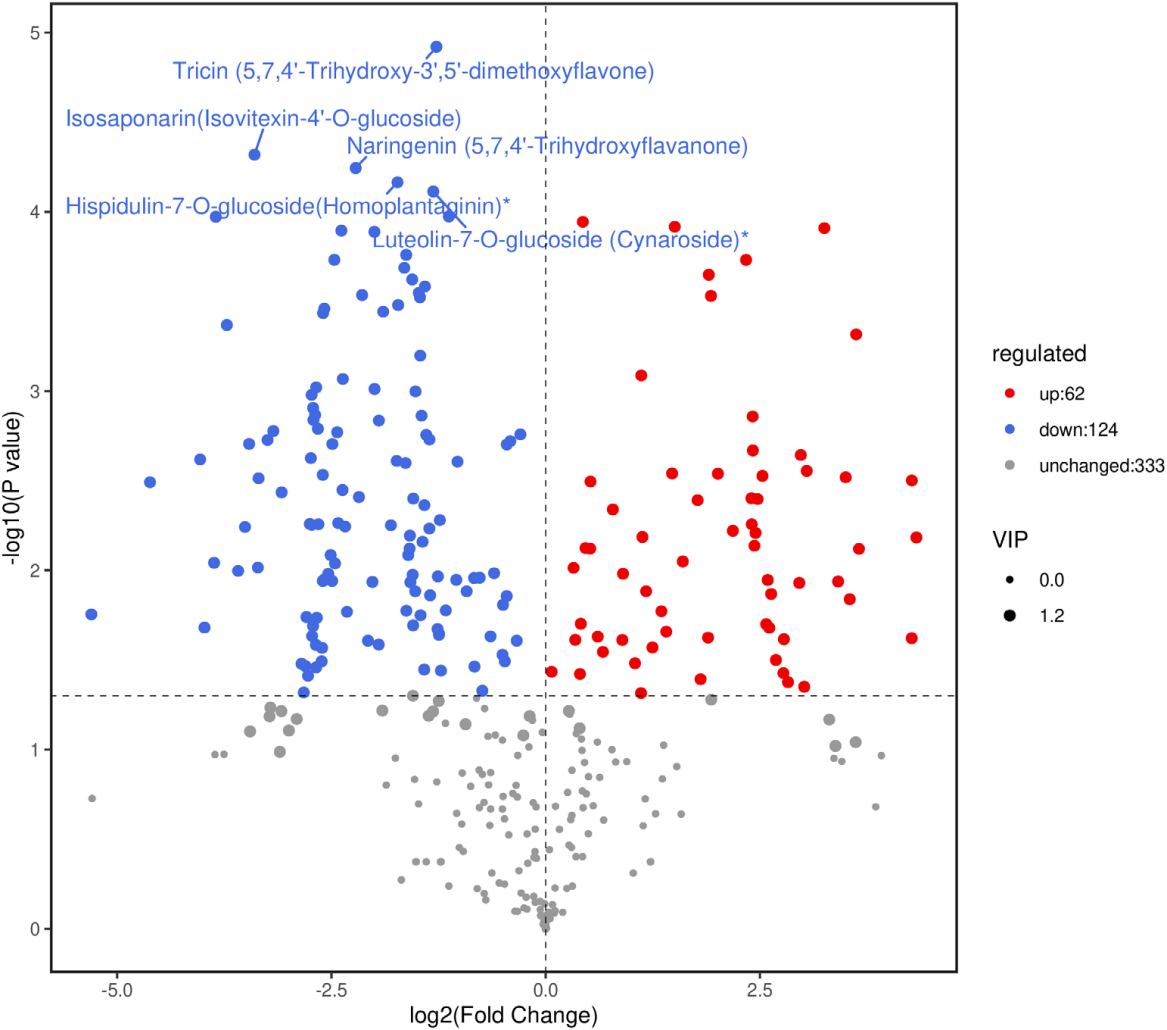
Differential analysis of phenolic metabolites between LC and AC. *Note:* LCore and ACore represent Longhua‐produced millet and Anze‐produced millet, respectively.

Systematic analysis identified 186 significantly different phenolic metabolites (Figure 3), with 124 upregulated in LC—particularly among flavonoids. The top five most differentially expressed metabolites (all higher in LC) included four flavonoids (Tricin, Isosaponarin, Naringenin, Cynaroside) and one flavonoid derivative (Homoplantaginin).

The 186 differential polyphenol metabolites were ranked based on *p*‐values, with the top five differentially expressed metabolites all showing higher levels in LC (please define LC if not previously mentioned). These included four flavonoids (Tricin, Isosaponarin, Naringenin, Cynaroside) and one flavonoid derivative (Homoplantaginin).

The most significantly different metabolite identified in the study was Cynaroside. Research by Lee et al. (2020) demonstrated that Cynaroside protected human periodontal ligament (hPDL) cells from LPS‐induced damage and inflammation via inhibition of NF‐κB activation. Additionally, Bouyahya et al. (2023) described Cynaroside's biological properties, which include antimicrobial, antioxidant, anticancer, and hepatoprotective effects.

The second most significant compound was Homoplantaginin. Feihua Wu's research indicated its potential application in preventing and treating insulin‐related endothelial dysfunction diseases. Additionally, Homoplantaginin has been shown to ameliorate liver injury (Qu et al. 2009).

Ranking third was Naringenin. Salehi et al. (2019) documented its diverse biological activities, including antioxidant, anti‐inflammatory, antiviral, and antitumor effects. Renugadevi and Prabu (2009) investigated naringenin's protective effects against cadmium‐induced kidney toxicity, demonstrating that its protective mechanism involves free radical scavenging and antioxidant capabilities.

Isosaponarin was studied by Nagai et al. (2010), who found it enhances collagen synthesis in human fibroblasts. Lu et al. (2022) demonstrated that Isosaponarin could inhibit Ca^2+^‐dependent PKC/SNAP‐25 and MARCKS pathways in synaptosomes, reducing the availability of synaptic vesicles and glutamate release, suggesting its potential as a therapeutic strategy for neurological disorders.

Tricin exhibits broad pharmacological activities (Zhou and Ibrahim 2010). Shalini et al. (2015) showed that tricin exerts anti‐inflammatory effects through mechanisms involving the TLR4/NF‐κB/STAT signaling cascade. Studies have identified tricin as a selective and effective inhibitor of both liver and pancreatic cancer cell lines, while showing no side effects on normal cells (Moheb et al. 2013). Additionally, tricin demonstrates anti‐influenza virus activity, improving weight loss and survival rates in mice infected with influenza A virus (Yazawa et al. 2011).

#### 3.2.3 KEGG Pathway Analysis of Differential Metabolites

During plant growth, multiple reactions and substances are required for coordinated regulation, constituting an extremely complex metabolic process. Overall assessments cannot be made solely based on the content level of any single substance. As the most fundamental biological process, metabolism requires further pathway analysis (Kanehisa 2017).

**FIGURE 4:**
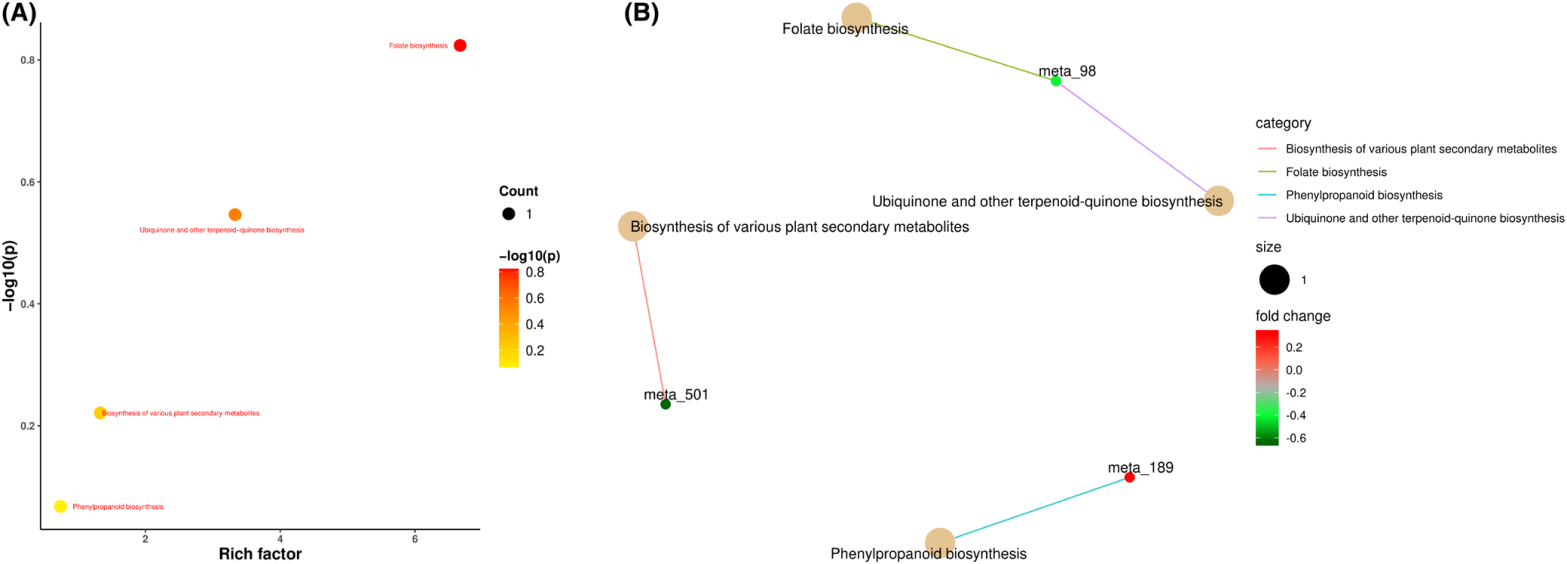
KEGG pathway enrichment analysis of differential metabolites. (A) Bubble plot of KEGG enrichment factors for differential metabolites. (B) Network plot of KEGG enrichment for differential metabolites. In the figure, light yellow nodes represent pathways, and the small nodes connected to them are specific metabolites annotated to those pathways.

KEGG, as a comprehensive database resource, provides crucial support for the association analysis of gene and genomic biological functions from molecular to higher levels (Kanehisa et al. 2016). Through KEGG database enrichment analysis of differential metabolites (Figure 4), a total of four metabolic pathways (*p* < 0.05) were identified, with three significantly differential metabolites annotated by KEGG. Among the four metabolic pathways, the most highly enriched was Folate biosynthesis, followed by Ubiquinone and other terpenoid‐quinone biosynthesis, Biosynthesis of various plant secondary metabolites, and Phenylpropanoid biosynthesis.

**FIGURE 5:**
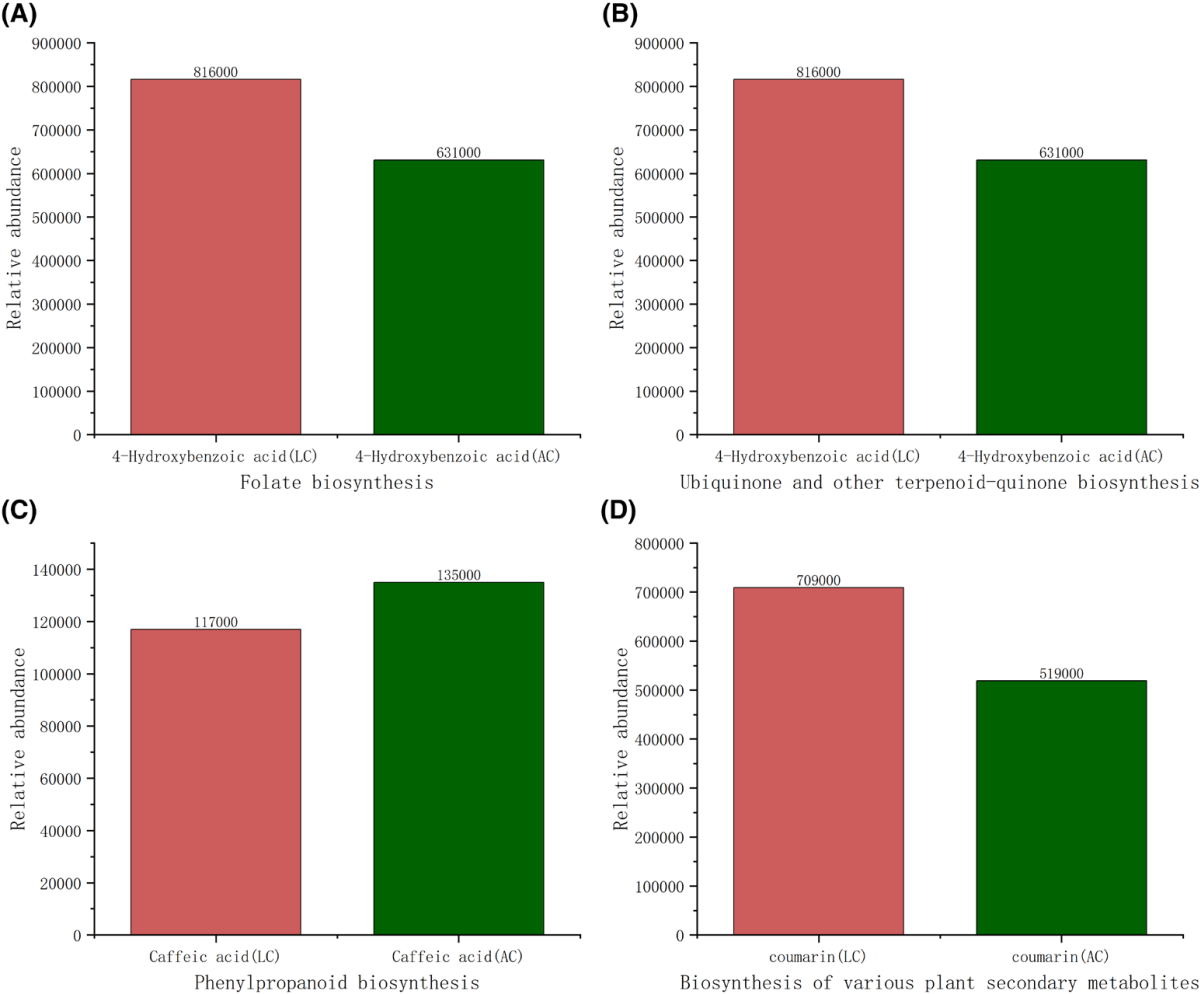
Relative abundance of plots of differential metabolites in four metabolic pathways (A–D). (A) Folate biosynthesis, (B) ubiquinone and other terpenoid‐quinone biosynthesis, (C) phenylpropanoid biosynthesis, (D) biosynthesis of various plant secondary metabolites. LC and AC represent Longhua‐produced millet and Anze‐produced millet, respectively.

As shown by the relative abundance (Figure 5), the differential metabolite 4‐hydroxybenzoic acid, which is enriched in the folate biosynthesis and ubiquinone/other terpenoid‐quinone biosynthesis pathways, exhibited a higher relative content in LC, being 1.2 times that of AC. The differential metabolite caffeic acid enriched in the phenylpropanoid biosynthesis pathway showed a slightly higher relative content in AC. Coumarin, a differential metabolite in the biosynthesis pathway of various plant secondary metabolites, demonstrated higher relative content in LC (1.3 times that of AC).

The most significantly enriched pathway, folate biosynthesis, primarily participates in metabolic processes involving cofactors and vitamins (Bertacine Dias et al. 2018). Tetrahydrofolate (THF) and its one‐carbon (C1) substituted derivatives are collectively referred to as folate (vitamin B9). THF serves as an indispensable cofactor in living organisms, acting as a carrier of C1 units in enzymatic reactions. It participates in the formation of purines, amino acids, pantothenic acid, and formyl methionyl‐transfer RNA, which in turn support cellular functions such as proliferation, mitochondrial respiration, and epigenetic regulation. Consequently, abnormalities in folate metabolism are causally linked to various diseases (Cossins 2000; Zheng and Cantley 2018). For instance, severe folate deficiency in the brain can lead to germline mutations in SLC46A1 or the production of folate autoantibodies. Moreover, insufficient folate intake during pregnancy increases the risk of neural tube defects. Cerebral folate deficiency, caused by folate receptor autoantibodies or germline mutations in FOLR1 or SLC46A1 (Watkins and Rosenblatt 2019), and insufficient folate intake around and during pregnancy have been associated with an increased risk of neural tube defects (Momb and Appling 2014). Humans and animals cannot synthesize folate and therefore rely on dietary sources, particularly from plants (Blancquaert et al. 2010; Scott et al. 2000). Folate exhibits significant antioxidant capacity, functioning as a ROS scavenger and mitigating oxidative damage induced by both biotic and abiotic stresses (Alsamadany et al. 2022).

A key metabolite in this pathway, 4‐hydroxybenzoic acid, is a natural phenolic compound. Research by Wang et al. (Sannino et al. 2018; Wang, Wang, et al. 2018) demonstrated that 4‐hydroxybenzoic acid could be applied as an effective inhibitor of histone deacetylase 6 (HDAC6) to reverse resistance to treatment in human breast cancer. Additionally, 4‐hydroxybenzoic acid possesses various biological activities, including neuroprotective (Winter et al. 2017) and antioxidant (Kou et al. 2024) effects.

### 3.3 Molecular Docking

Using AutoDock Vina software, the 186 differential metabolites detected in the two millet samples were individually docked with 21 key antioxidant proteins (ALDH2, BGN, CAT, CBS, CEL, SIRT1, RPE, etc.). The results showed that 96 polyphenolic compounds exhibited good docking performance and potential antioxidant activity (binding energy between compound ligands and protein receptors < −5 kJ/mol). When ranked by binding energy, compounds including Homoplantaginin, 6‐Hydroxykaempferol‐7‐O‐glucoside, Luteolin‐3′‐O‐glucoside, and Cynaroside demonstrated binding energies ≤ −11 kJ/mol, all of which were highly expressed in Longhua‐produced millet (as shown in Table 1).

**TABLE 1:** Binding energies of effective compounds with core targets.

Compound	Target	Binding energy (kJ/mol)
Homoplantaginin	CAT	−12.8
6‐Hydroxykaempferol‐7‐O‐glucoside	CAT	−12.3
Luteolin‐3′‐O‐glucoside	CAT	−11.9
Cynaroside	CAT	−11.6
Chrysoeriol‐7‐O‐gentiobioside	SIRT1	−11.5
3,3′‐Di‐O‐Methylellagic acid 4′‐glucoside	RPE	−11.4
Chrysoeriol‐7‐O‐glucoside	CAT	−11.4
Apigenin‐7‐O‐Gentiobioside	BGN	−11.3
Diosmetin‐7‐O‐glucoside	CAT	−11.3
Iristectorin A	CAT	−11.1

**FIGURE 6:**
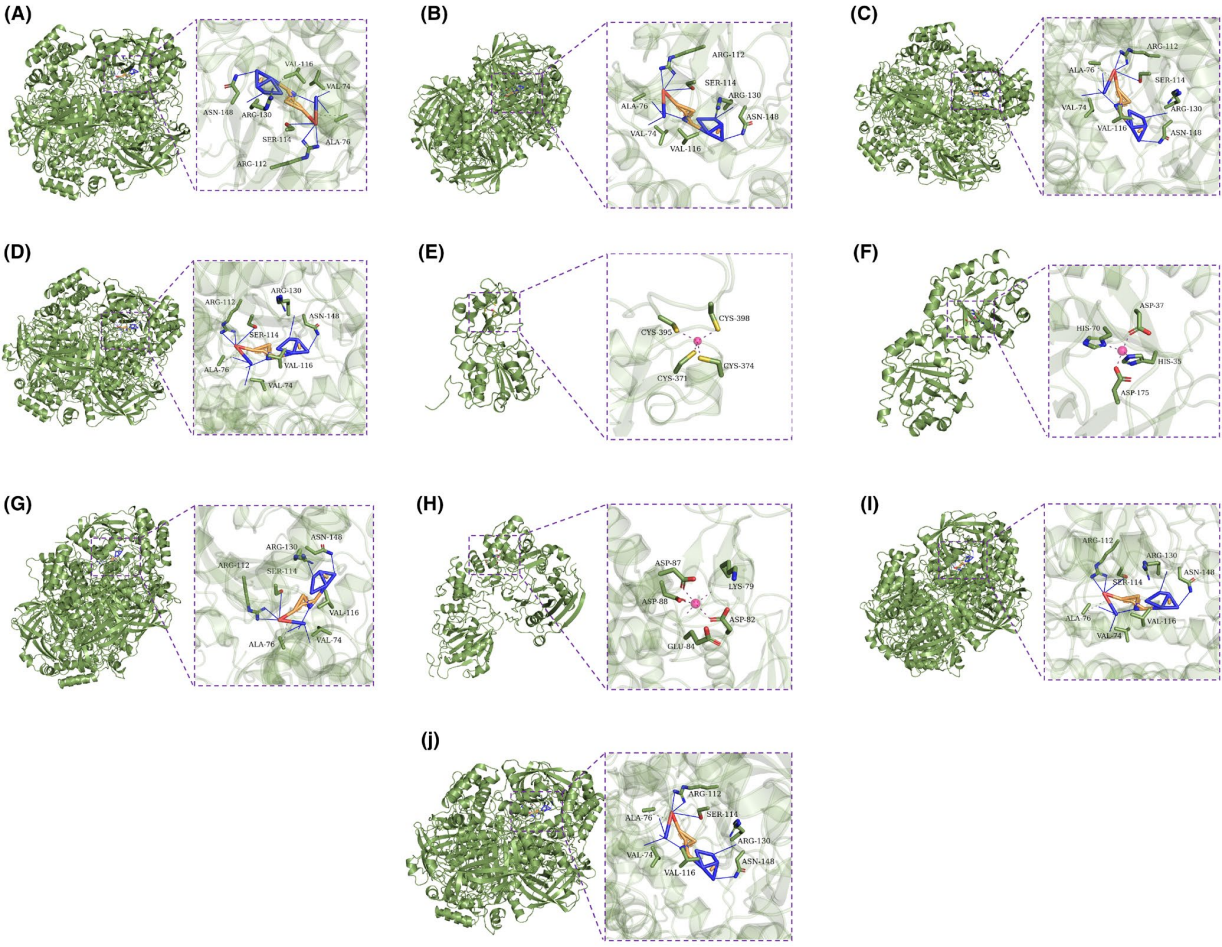
Molecular docking results diagram (A): Homoplantaginin‐CAT; (B): 6‐Hydroxykaempferol‐7‐O‐glucoside‐CAT; (C): Luteolin‐3′‐O‐glucoside‐CAT; (D): Cynaroside‐CAT; (E): Chrysoeriol‐7‐O‐gentiobioside ‐SIRT1; (F): 3,3′‐Di‐O‐Methylellagic acid 4′‐glucoside‐RPE; (G): Chrysoeriol‐7‐O‐glucoside‐CAT; (H): Apigenin‐7‐O‐Gentiobioside‐BGN; (I): Diosmetin‐7‐O‐glucoside‐CAT; (J): Iristectorin A ‐CAT.

In PyMOL software, the complexes of the aforementioned proteins with small molecules were visualized and analyzed, with partial results shown in Figure 6. The analysis revealed the following interactions:

For Homoplantaginin–CAT complex: Hydrogen bonds formed with ALA‐76, VAL‐74, ARG‐112, SER‐114, ARG‐130, and ASN‐148 of CAT. Hydrophobic interactions with ALA‐76 and VAL‐116 of CAT.

For 6‐Hydroxykaempferol‐7‐O‐glucoside–CAT complex: Hydrogen bonds formed with VAL‐74, ALA‐76, ARG‐112, SER‐114, ARG‐130, and ASN‐148 of CAT. Hydrophobic interactions with ALA‐76 and VAL‐116 of CAT.

For Luteolin‐3′‐O‐glucoside–CAT complex: Hydrogen bonds formed with ARG‐112, SER‐114, ARG‐130, ALA‐76, VAL‐74, and ASN‐148 of CAT; hydrophobic interactions with ALA‐76 and VAL‐116 of CAT.

For Cynaroside–CAT complex: Hydrogen bonds formed with ARG‐130, SER‐114, ASN‐148, ARG‐112, ALA‐76, and VAL‐74 of CAT. Hydrophobic interactions with ALA‐76 and VAL‐116 of CAT.

For Chrysoeriol‐7‐O‐gentiobioside–SIRT1 complex: Hydrogen bonds formed with ALA‐262, ARG‐274, HIS‐363, SER‐441, and SER‐442 of SIRT1. Hydrophobic interactions with ALA‐262, PHE‐273, and GLN‐345 of SIRT1.

For 3,3′‐Di‐O‐Methylellagic acid 4′‐glucoside–RPE complex: Hydrogen bonds formed with LEU‐12, ASN‐13, ASN‐46, HIS‐51, GLY‐149, GLY‐176, GLY‐197, and SER‐198 of RPE. Metal complexes formed with HIS‐35, ASP‐37, HIS‐70, and ASP‐175 of RPE.

For Chrysoeriol‐7‐O‐glucoside–CAT complex: Hydrogen bonds formed with VAL‐74, ALA‐76, ARG‐112, SER‐114, ARG‐130, and ASN‐148 of CAT. Hydrophobic interactions with ALA‐76 and VAL‐116 of CAT.

For Apigenin‐7‐O‐Gentiobioside–BGN complex: Metal complexes formed with LYS‐79, ASP‐82, GLU‐84, ASP‐87, and ASP‐88 of BGN. Hydrogen bonds formed with SER‐34, ARG‐191, ASP‐252, TYR‐289, GLY‐292, TRP‐314, GLU‐317, ASP‐350, and ASN‐353 of BGN.

For Diosmetin‐7‐O‐glucoside–CAT complex: Hydrogen bonds formed with VAL‐74, ALA‐76, ARG‐112, SER‐114, ARG‐130, and ASN‐148 of CAT. Hydrophobic interactions with ALA‐76 and VAL‐116 of CAT.

For Iristectorin A–CAT complex: Hydrogen bonds formed with VAL‐74, ALA‐76, ARG‐112, SER‐114, ARG‐130, and ASN‐148 of CAT. Hydrophobic interactions with ALA‐76 and VAL‐116 of CAT. These interactions enable the formation of stable protein–compound complexes with favorable binding activity and structural stability.

### 3.4 Validation of Antioxidant Results for Phenolic Monomers

**FIGURE 7:**
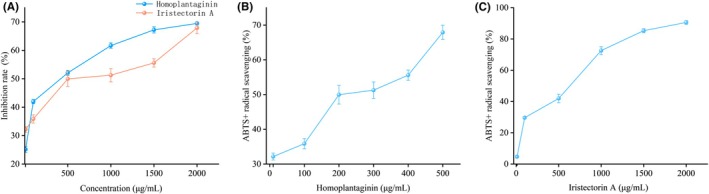
Antioxidant activity of phenolic monomers. (A) Anti‐lipid peroxidation activity; (B, C) ABTS scavenging activity.

Inhibition of Fe^2+^‐induced lipid peroxidation in egg yolk lipoproteins: As shown in Figure 7A, Homoplantaginin and Iristectorin A exhibited concentration‐dependent inhibition rates within the range of 10–2000 μg/mL. At 2000 μg/mL, Homoplantaginin achieved a maximum inhibition rate of 74.85% ± 0.38%, while Iristectorin A reached 69.48% ± 0.56%.

ABTS radical scavenging assay: Figure 7B,C demonstrate that the inhibition rates of Homoplantaginin and Iristectorin A increased proportionally with concentration. Homoplantaginin attained its maximum inhibition rate of 67.9% ± 2.05% at 500 μg/mL; whereas, Iristectorin A achieved 90.57% ± 1.23% at 2000 μg/mL.

## 4 Discussion and Conclusions

This study systematically integrated widely targeted metabolomics, molecular docking technology, ultrasound‐assisted extraction techniques, and in vitro antioxidant activity evaluation systems. The aim was to comprehensively elucidate the enrichment patterns of phenolic metabolites and their antioxidant mechanisms in foxtail millet from Longhua and Anze regions of Shanxi Province.

The results demonstrate that millet extracts from both regions are rich in polyphenolic components. These polyphenols exhibit a concentration‐dependent enhancement in both DPPH radical scavenging capacity and inhibition of Fe^2+^‐induced lipid peroxidation in egg yolk lipoproteins, demonstrating significant antioxidant activity and representing high‐quality natural antioxidant sources (Shahidi and Chandrasekara 2013). Notably, compared to Anze millet (AC), Longhua millet (LC) displays superior antioxidant capacity. While the AC extract required a concentration of 200 μg/mL to reach the maximum DPPH scavenging rate and lipid peroxidation inhibition, the LC extract achieved comparable maximum effects at just 80 μg/mL.

Through widely targeted metabolomics analysis, 186 significantly different phenolic metabolites were identified between LC and AC, with 124 differentially accumulated polyphenolic metabolites showing high accumulation characteristics in LC, primarily flavonoids. This finding reveals that the geographical environment may significantly influence millet polyphenol metabolic profiles: the semi‐mountainous terrain of Longhua Town (with an average annual temperature of 10°C–12°C and approximately 2400 h of sunshine) enhances flavonoid biosynthesis (e.g., tricin, naringenin), while high UV radiation stimulates the accumulation of phenolic compounds as photoprotective agents. The mountain cinnamon soil type (pH 6.5–7.0) in Longhua Town facilitates mineral absorption (e.g., Fe^2+^, Zn^2+^), which serves as cofactors for P450 monooxygenases involved in flavonoid glycosylation. These environmental factors may represent key contributors to the differential polyphenol metabolites between the two regions.

Based on *p*‐value ranking, key metabolites such as Tricin, Isosaponarin, Naringenin, Homoplantaginin, and Iristectorin A were identified as making significant contributions to the antioxidant properties of millet from both regions. Metabolic pathway analysis demonstrated that the folate biosynthesis pathway plays a crucial regulatory role in millet polyphenol metabolism.

Molecular docking revealed that both Homoplantaginin and Iristectorin A, which exhibit high expression characteristics in LC, demonstrated strong antioxidant binding activity. In vitro antioxidant experiments further showed that both compounds display significant antioxidant activity at low concentrations, validating the molecular docking results. This suggests that Longhua millet may exert its antioxidant effects through phenolic compounds such as Homoplantaginin and Iristectorin A by acting on antioxidant genes including CAT.

The series of research findings demonstrates that the antioxidant activity of millet phenolic metabolites is influenced by geographical factors. The superior antioxidant capacity of LC compared to AC is primarily associated with its abundant flavonoid content.

## Author Contributions

**Xuan Zhao:** conceptualization (lead), data curation (lead), formal analysis (lead), investigation (lead), methodology (lead), project administration (lead), software (lead), validation (lead), visualization (equal), writing – original draft (supporting), writing – review and editing (lead). **Luhong Yang:** funding acquisition (supporting), project administration (supporting), resources (supporting), supervision (supporting). **Yaoru Li:** conceptualization (equal), investigation (equal), supervision (equal).

## Ethics Statement

The authors have nothing to report.

## Conflicts of Interest

The authors declare no conflicts of interest.

## Data Availability

The data that support the findings of this study are available from the corresponding author upon reasonable request.
